# Pollination success increases with plant diversity in high-Andean communities

**DOI:** 10.1038/s41598-021-01611-w

**Published:** 2021-11-11

**Authors:** Sabrina S. Gavini, Agustín Sáez, Cristina Tur, Marcelo A. Aizen

**Affiliations:** 1 Grupo de Ecología de la Polinización-Instituto de Investigaciones en Biodiversidad y Medio Ambiente (INIBIOMA), CONICET-Universidad Nacional del Comahue, Quintral 1250, 8400 San Carlos de Bariloche, Rio Negro Argentina; 2 Unaffiliated, Palma de Mallorca, Spain

**Keywords:** Community ecology, Ecosystem ecology

## Abstract

Pollinator-mediated plant–plant interactions have traditionally been viewed within the competition paradigm. However, facilitation via pollinator sharing might be the rule rather than the exception in harsh environments. Moreover, plant diversity could be playing a key role in fostering pollinator-mediated facilitation. Yet, the facilitative effect of plant diversity on pollination remains poorly understood, especially under natural conditions. By examining a total of 9371 stigmas of 88 species from nine high-Andean communities in NW Patagonia, we explored the prevalent sign of the relation between conspecific pollen receipt and heterospecific pollen diversity, and assessed whether the incidence of different outcomes varies with altitude and whether pollen receipt relates to plant diversity. Conspecific pollen receipt increased with heterospecific pollen diversity on stigmas. In all communities, species showed either positive or neutral but never negative relations between the number of heterospecific pollen donor species and conspecific pollen receipt. The incidence of species showing positive relations increased with altitude. Finally, stigmas collected from communities with more co-flowering species had richer heterospecific pollen loads and higher abundance of conspecific pollen grains. Our findings suggest that plant diversity enhances pollination success in high-Andean plant communities. This study emphasizes the importance of plant diversity in fostering indirect plant–plant facilitative interactions in alpine environments, which could promote species coexistence and biodiversity maintenance.

## Introduction

Understanding the mechanisms that drive species coexistence is challenging, especially given the underlying belief that interspecific competition is the overriding ecological force determining diversity^1^. A growing number of studies, however, have shown that plant–plant interactions can range from competitive to facilitative^2–5^ and animal-mediated interactions are not an exception^6–8^. Given that most flowering plant species are animal-pollinated^9^ and generalization in plant-pollinator interactions is widespread^10,11^, pollinator sharing is common among co-flowering plants. Shared pollinators move pollen not only between conspecific but also among heterospecific flowers, and as a result interspecific pollen transfer is ubiquitous in communities worldwide^12^. Interspecific pollen transfer has certainly been seen as one of the mechanisms underlying competition among plants for pollinators^13,14^, notwithstanding it can also reflect facilitation^7,15–18^. Ultimately, the study of interspecific pollen transfer can be useful to better understand the role of pollinator-mediated plant–plant interactions as drivers of plant species coexistence and diversity.

Pollen transfer among flowers from different species is particularly common in natural communities^12^ and several recent studies show that heterospecific pollen loads on stigmas, albeit highly variable, can be large and diverse^7,19–21^. Commonly, more than 50% of the flowers of outcrossing plant species receive some heterospecific pollen, and in amounts that constitute from ~ 1 to 70–80% of the total pollen load deposited on the stigmas^19–22^. Moreover, heterospecific pollen grains can come from a single or multiple donor species^7,20,23^. This substantial variation within and among species in both the magnitude and diversity of heterospecific pollen loads may be due to several factors such as differences in plant specialization, flower symmetry and size, flower lifespan, floral display, and associated pollinator group^12,22,24^. However, beyond all these intrinsic factors, the size and diversity of pollen loads on flowers’ stigmas might reflect local plant community diversity, and be a consequence of the processes triggered by several species flowering together.

The outcome of pollinator sharing on plant pollination success can range from negative to positive^8^. Specifically, heterospecific pollen deposited by shared pollinators can have negative effects when it interferes either mechanically or physiologically with conspecific pollen receipt and performance (i.e., pollen germination and pollen tube growth), ultimately decreasing seed production^13,14,19,25–28^. Nevertheless, heterospecific pollen deposition does not necessarily translate into negative effects. Some studies have found neutral effects of heterospecific pollen transfer on reproductive success in recipient plants^29–31^. On the other hand, Tur et al.^7^ showed that for many species increases in heterospecific pollen receipt were accompanied by increases in conspecific pollen deposition and germination on stigmas, and proposed that the relation between conspecific and heterospecific deposition on stigmas can be used as a proxy for the plant cost–benefit balance derived from pollinator sharing. After all, the species diversity and composition of the stigmatic pollen community provide information not only on patterns of pollinator movement among co-occurring flowering plant species, but also on the functional consequences of pollinator sharing^7,14^.

All else being equal, a positive relation between conspecific pollen grains and heterospecific pollen richness on flowers’ stigmas could be a reflection of increased shared pollinator attraction by different co-flowering species^15,16,18,32–36^ and increasing occurrence of species that act as pollinator “magnets”^37^. The outcome of interspecific pollen receipt is, however, highly context-dependent as it can vary according to the identity and phylogenetic relatedness of the donor and recipient species involved^19,27,28,38,39^. Other extrinsic factors that can influence this outcome are the timing of heterospecific pollen arrival on stigmas^40^, the diversity of the heterospecific pollen load^27^, and the environmental conditions and disturbances to which interacting plant species are subjected^7,41–43^. In any event, the basic questions of how conspecific pollen deposition relates to the diversity of the heterospecific pollen load and whether these two pollination-related variables, i.e. conspecific pollen receipt and stigmatic pollen diversity, reflect plant community diversity have remained unexplored.

It has been proposed that in severe environments facilitative interactions are paramount^2,44,45^. This seems to be the case of alpine ecosystems that are subject to frequent disturbances and intense abiotic stress^46^. In these harsh environments, characterized by extremely low and widely-ranging temperatures, high evapotranspiration, intense UV-radiation and nutrient-poor skeletal soils, nurse plants facilitate the establishment and survival of many plant species^47–49^. By examining the relation of conspecific to heterospecific pollen receipt in diverse Andean communities, Tur et al*.*^7^ concluded that pollinator-mediated facilitation could also be widespread in these poor-pollinator environments. Yet these authors did not evaluate the pollinator-mediated facilitative role of plant diversity. Here, we assessed the effect of plant diversity on pollination success, estimated as the receipt of conspecific pollen, in nine high-Andean communities of Northwestern Patagonia. Accordingly, we addressed the following questions: (1) What is the prevalent sign of the relation of conspecific pollen receipt to the number of donor species in the stigmas within and across communities? (2) Does the incidence of different species-specific outcomes (i.e., positive, neutral, or negative) change along the altitudinal gradient? (3) How do conspecific pollen receipt and pollen diversity on the stigmas relate to the diversity of the flowering community?

## Results

We counted a total of 166,945 pollen grains (159,373 CP and 7572 HP grains) on 9371 stigmas across the nine communities (Fig. S1), representing 88 high-Andean species from 27 angiosperm families (Table S1). The most frequent, and thus most sampled family was Asteraceae (39 spp., 44%) followed by Fabaceae (6 spp., 6.8%) and Caprifoliaceae (5 spp., 5.6%). For the rest of the families, the number of sampled species varied from one to four. Total pollen load per stigma was extremely variable ranging from 0 to 520 grains (range: CP = 0–520; HP = 0–77). The highest HP load, 77 pollen grains, was observed on a stigma of *Euphrasia meiantha* (Orobanchaceae) at 1600 m in Catedral mountain. The highest number of HP donor species identified on a single stigma was six, observed in *Valeriana carnosa* (Caprifoliaceae) at 1600 m in Challhuaco mountain. Stigmas without HP were common at all communities (see Fig. S1), additionally the highest diversity of HP on individual stigmas was observed in Challhuaco mountain at all elevations (Fig. S1). We found HP in 19.5% (n = 1829) and CP in 86.2% (n = 8082) of all stigmas analyzed, whereas we did not observe pollen grains in 12.9% of the stigmas (n = 1210). The percent of stigmas without pollen grains was 10.9% at 1600 m, slightly increasing to 14.1% and 13.7% at 1800 and 2000 m, respectively. HP accounted for (mean ± SE) 5.2 ± 0.17% of the total pollen load on stigmas across all flowers sampled; therefore, stigmas presented mostly CP (94.8 ± 0.17%). Flowers received, on average, 0.81 ± 0.03 HP grains per stigma, a value that decreased with altitude (1 ± 0.06, n = 3210 at 1600 m; 0.8 ± 0.05, n = 3947 at 1800 m; and 0.5 ± 0.06 HP grains, n = 2214 stigmas at 2000 m).

Among stigmas with HP (n = 1829), 78% (n = 1424) received HP from a single donor species, 18% (n = 337) from two donor species, and ca. 4% (n = 68) from three or more different donor species. The percentage of stigmas with HP from multispecies (i.e., from > 1 heterospecific donor species) decreased with altitude (5.7, 4.2 and 2.8% at 1600, 1800, and 2000 m, respectively). The number of plant species in flower sampled in each community at a given time averaged nine species, ranging between one and 24 species. Mean number of species in flower sampled in a community at a given time was highest at 1800 m (10.1 species, range = 2–24 species), followed by 1600 m (9.4 species, range = 1–17 species), whereas the lowest mean species numbers were found at 2000 m (7.4 species, range = 3–11 species).

**Figure 1:**
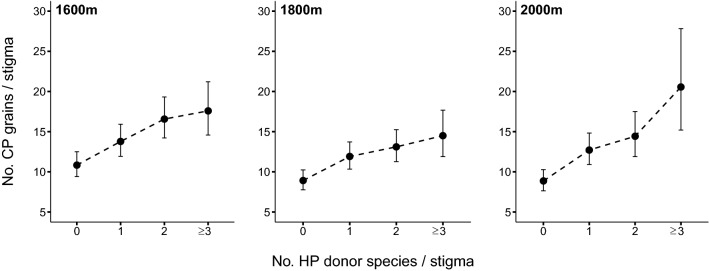
Adjusted mean values (± 1 SE) of conspecific pollen (CP) receipt from the fitted global model for each altitude (1600, 1800 and 2000 m) and level of heterospecific pollen (HP) richness (i.e., number of HP donor species/stigma). For estimating these adjusted means, model was re-run considering HP richness as a factor rather than as a continuous variable, thus “0” category represents the absence of HP, “1” the presence of HP from one donor species, “2” HP from two species, and “= or > 3” HP from three or more donor species.

There was a consistent increase in CP receipt with increasing number of HP donors present in the HP load at all altitudes (Fig. 1). The overall GLMM showed that the number of CP grains on the stigma was associated positively with HP richness (β ± SE = 0.21 ± 0.026, z = 8.007, *P* < 0.0001) independently of the positive effect of the HP load size (β ± SE = 0.0084 ± 0.0028, z = 2.975, *P* = 0.003). The standardization of these two predictors showed that the effect of HP richness was 4.4 times greater than the effect of HP abundance (β′ ± SE = 0.117 ± 0.015 vs. 0.026 ± 0.009, respectively). Neither altitude nor its interaction with HP richness had a significant effect on CP receipt (Table S2). Overall, mean increase of CP deposition associated with an increase of HP from only one to three or more donor species was 37.1% (Fig. 1).

**Figure 2:**
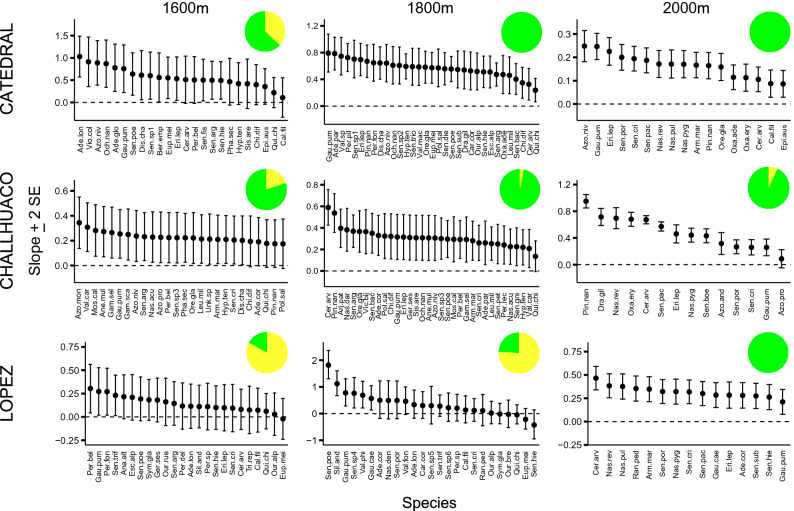
Estimated slopes (± 2 SE) from the relation between heterospecific pollen (HP) richness receipt and conspecific pollen (CP) deposition on stigmas for each receptor plant species at the nine high-Andean communities studied ordered according to mountain (rows) and altitude (column). Coloured pie charts represent the percentage of species which experienced an overall facilitative (green) or neutral (yellow) effect from sharing pollinators with other plants in the community.

The relation between the number of HP donor species and CP receipt for individual species was either neutral or positive but never negative (Fig. 2). In two communities (Cerro López at 1600 and 1800 m), neutral relations were more common (i.e., the 95% confidence interval of the species-specific slope of the relation of number of CP grains to HP richness overlapped with 0 in > 50% of the species) than positive relations, whereas in the other seven communities positive relations were more common than neutral relations (Fig. 2). Moreover, as seen in Fig. 2, there was an increase in the percentage of species exhibiting positive relations between CP receipt and HP richness with increasing altitude. Specifically, the percentage of species, averaged across the three mountains, showing evidence of facilitation was 53% at 1600 m, 74% at 1800 m, and 98% at 2000 m (Chi-square test, *X*^2^ = 54, df = 2, *P* < 0.001).

**Figure 3:**
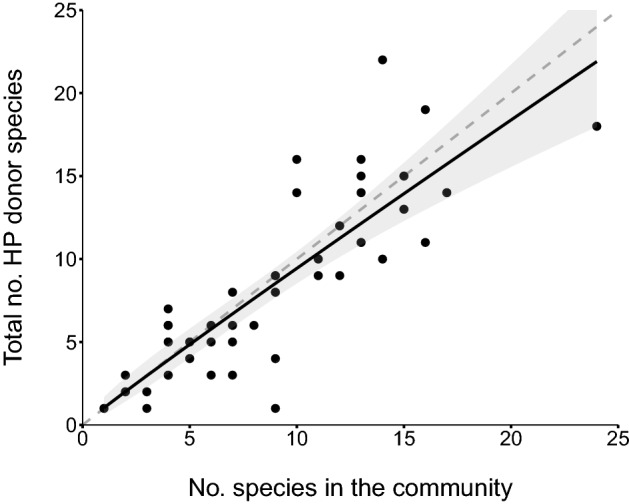
Number of distinct heterospecific pollen (HP) donor species found across all sampled stigmas in a community at a given sampling date as a function of the number of co-flowering plant species. Model predicted values (solid line) with 95% confidence intervals (shaded area) are shown. The dashed line (intercept = 0 and slope = 1) represents the theoretical expectation of the total cumulative number of pollen donor species being equal to the number of plant species in bloom in the community.

**Figure 4:**
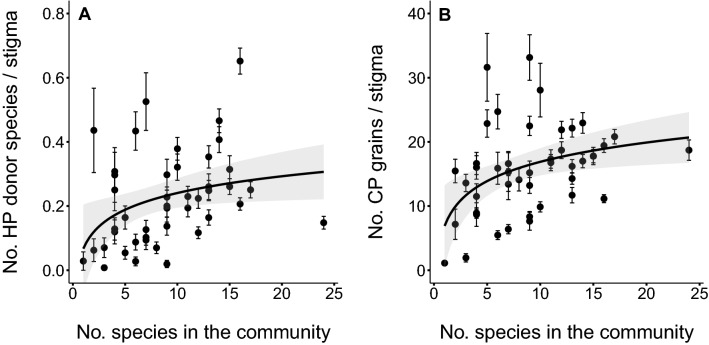
Mean ± SE values for the number of (**A**) heterospecific pollen (HP) donor species per stigma and (**B**) conspecific pollen (CP) grains per stigma as a function of the total number of plant species in bloom present in the community. Model predicted values (solid line) and 95% confidence intervals (shaded area) are shown. Each point represents the mean across all species within each combination of mountain, altitude and sampling date.

Rarefaction analysis of sampling completeness (Fig. S2a) showed that the Chao-2 asymptotic estimate of the expected number of HP donor species in each community × time combination correlated almost perfectly with the observed total number of HP donor species (*r* = 0.97, *P* < 0.0001, Fig. S2a,b). The observed values captured at least 90% of the expected number of HP donor species in 34 out of the 44 temporal communities sampled. In turn, the observed number of HP donor species found on the stigmatic pollen loads reflected the number of plant species in bloom in each community at a given time. In fact, the fitted regression of the total number of distinct pollen donor species identified across the stigmas sampled in each community at a given time indicates that the number of HP donors sampled in the flower’s stigmas basically equals the number of species blooming in the community (β ± SE = 0.96 ± 0.10, z = 9.29, *P* < 0.0001; Fig. 3). In addition, we found low dissimilarity between the species represented in the HP loads and the species flowering in the community (mean βsor ± SE = 0.32 ± 0.03, a value much closer to 0 than 1). Although values vary across communities (Table S3), we found an increase in dissimilarity with increasing altitude (*X*^2^ = 9.5, df = 2, *P* = 0.0087; Fig. S3). On average, both limited species turnover and species loss contributed similarly to explain this low dissimilarity in species composition between assemblages (mean ± SE, βsim = 0.20 ± 0.04 and βnes = 0.12 ± 0.02; Fig. S3). Finally, mean HP richness and CP amount per stigma increased monotonically but in a decelerating fashion with the number of plant species in bloom in the community (*X*^2^ = 7.730, df = 1, *P* = 0.005; and *X*^2^ = 19.38, df = 1, *P* < 0.0001; Fig. 4A,B). Both HP richness and CP load increased with the number of flowering plants in the community at similar rates (β ± SE = 0.45 ± 0.16 vs. 0.54 ± 0.12 for HP richness and CP receipt, respectively).

## Discussion

Direct plant–plant facilitation has been proposed as a prevailing force structuring communities and fostering diversity under severe environmental conditions like those characterizing arid and alpine ecosystems^2,5,44,48–50^. The importance of facilitation in severe ecosystems may extend to indirect plant–plant interactions, particularly to those mediated by mutualistic partners such as pollinators^7,17,33,51^. Contrary to this view, most pollinator-mediated interactions have been characterized as competitive^13,14,19^. Such competition has been proposed as an explanation for increased pollen limitation in highly diverse communities^52^. Moreover, the relation between plant diversity and the sign and magnitude of pollinator-mediated plant–plant interactions has remained largely unexplored. Using a community-wide perspective, we assessed the relation of CP receipt to the number of HP donors in the flowers’ stigmas, the signature of pollinator sharing among plants, and we explored how both variables relate to plant community diversity. Our results show that stigmas receiving pollen grains from more heterospecific donors also received more CP grains. This positive relation prevailed in most of the communities studied; further, the proportion of species showing it increased with altitude. Additionally, the number of species represented in the stigmatic HP load and the amount of CP receipt increased asymptotically with the increasing number of co-flowering plant species in the community. Overall, these findings provide strong, albeit indirect, evidence that facilitation through shared pollinator attraction characterized the high-Andean communities studied, highlighting the importance of plant diversity for this facilitative process.

### The community-wide approach: advantages and limitations

Our understanding of indirect pollinator-mediated interactions is principally based on studies involving pairs or a reduced number of plant species [^25–27,38,39,41^, but see^42,53^]. The reason for this is that simple systems are amenable to experimental manipulation. However, this approach excludes a whole-community perspective, which is needed to evaluate the relevance of pollinator-mediated interactions for biodiversity maintenance. Therefore, the development of new conceptual frameworks and practical alternative approaches have become imperative to assess the consequences of pollinator sharing at the community scale. One useful approach is a detailed description of patterns of conspecific and heterospecific pollen transfer within diverse plant communities, which allows the construction of pollen transfer networks^7,20,54^ and the detection of potential “magnet” species^7,55^. Indeed, the description of pollen loads on stigmas has shown to be a powerful tool for assessing the sign and strength of plant–plant interactions driven by pollinator sharing at a community scale^7^. This method, however, has been criticized owing to limitations related to the lack of a measure of plant fitness (i.e., seed production) and the role of potential confounding factors (e.g., pollinator activity) that need to be controlled experimentally or, at least, statistically^56^. Here, we control statistically for confounding factors that could vary seasonally by including time as a proxy factor in most analyses. Also, the interpretation of our results under the pollinator-mediated facilitation hypothesis is strengthened because of the robust connection of CP and HP with actual plant community diversity. In any event, experimental field assays are necessary to truly demonstrate whether larger CP loads associated with diverse HP loads indeed are linked to greater fitness among the recipient plants, although this is highly likely given the high pollinator dependence of the study plant communities despite pollinator scarcity^57–59^.

### Low magnitude of interspecific pollen transfer but from multiple donors

Pollen loads on stigmas contained mostly CP, whereas HP occurred at relatively low levels as it was found in only ~ 20% of the flowers, representing, on average, 5% of the total pollen load deposited. This is partially consistent with other studies that also found low deposition of HP in natural communities^23,40,60^. Despite low incidence and magnitude of HP deposition, stigmatic pollen loads including pollen from two or more HP donor species were not infrequent. The commonness of plant species receiving pollen from many other species has been reported in other studies^20,21,23^. For instance, an analysis performed in a highly diverse alpine meadow of Hengduan mountains, China, found that plant species received pollen from an average of ~ 7 heterospecific donor species^20^ and individual stigmas from an average of ~ 2 heterospecific pollen donor species^22^. These findings indicate that, despite much pollinator constancy^30^, interspecific flower visits during single foraging bouts are frequent in natural communities. In the case of mainly bumble-bee pollinated communities, like ours, this can relate to the “majoring” and “minoring” behavior of these bees^61^. Thus, while individual bumble bee foragers commonly specialize on the flowers of a single “major” species, they commonly include a few visits to one or more alternative “minor” species. Moreover, interspecific visits can be more common in stressful environments since it has been observed that the feeding niche-breadth of bees increases with altitude^62^.

### The relation of conspecific pollen to the number of heterospecific pollen donors

HP can interfere mechanically or physiologically with the deposition and germination of CP on a flower’s stigma, as well as with pollen tube growth, ovule fertilization, and seed production^13,19,25,28^. In fact, HP load diversity is known to contribute negatively to recipient post-pollination outcomes^27^. In the current study, the low abundance of HP on flowers’ stigmas is unlikely to affect the pollination and post-pollination performance of CP. On the contrary, Tur et al*.*^7^ previously reported on a positive relation between HP abundance and CP deposition and germination in this system, indicating that any negative effect related to HP receipt is overcome by the positive effect of co-occurring heterospecific flowers on pollinator attraction. Here, we further show that CP receipt increase with the number of HP donor species on stigmas, independently of the total amount of foreign pollen grains. The relation of CP with the number of pollen donor species was much stronger than with the quantity of HP grains itself. This finding supports the view of the facilitative role of diverse communities of co-flowering plants on each other species’ pollination. Because the relatively high flower constancy of bumble bees^63^, the increasing number of species acting as pollen donors represented in stigmas in diverse communities is probably the result of an increasing number of visits by different bumble bee individuals, majoring on the same focal species but each one minoring on different plant species^64^.

An increasing proportion of species showed a positive relation between CP receipt and HP richness along the altitudinal gradient, a result which provides support for the *Stress Gradient Hypothesis*. This hypothesis states that the importance of facilitation in community structure and diversity maintenance increases with environmental stress^2,3,44,45^. Even though this hypothesis was first proposed for understanding the prevalence of facilitative direct plant–plant interactions in alpine and arid environments^48–50^, it can be extended to indirect plant–plant interactions as those mediated by shared pollinators^7,17,33,37^. In the context of our study, and others^43^, we can incorporate plant diversity as a key factor in this hypothesis. After all, pollinator visitation frequency in multispecies communities of co-flowering plants represents a balance between the positive effect of higher pollinator recruitment with the addition of a new co-flowering species to the patch and the negative effect of a total number of visits becoming diluted among an increasing number of flowers^65^. A positive relation between CP and HP diversity, a reflection of higher per-flower pollinator visitation, implies that, on average, the pollinator recruitment effect surpasses the visit dilution effect. The relevance of this diversity-dependent pollinator recruitment effect is likely to increase as the environment become more stressful and increasingly devoid of both vegetation and pollinators, as it happens along altitudinal gradients^66,67^.

### The community diversity connection

Our results showed that the overall number of pollen donor species found in pollen loads deposited per stigma reflected the surrounding plant diversity. First, the cumulative number of different HP donors found on flowers’ stigmas basically equals the number of species in flower in the plant community (Fig. 3). Even the resemblance in terms of species composition between the deposited heterospecific pollen and plant community was remarkable. The remaining dissimilarity could be explained by the fact that some species are, probably because of morphology or limited attraction, poor pollen donors to other species^7,20,54^. Also, bumble bees and other pollinators can fly long distances and bring pollen from plants several hundred meters away^68^, thus transferring pollen from plant species not present in our spatially-limited plant communities. This latter effect seems to accentuate with altitude (Fig. S3), as vegetation becomes less diverse and sparser^49^. Second, the diversity of the plant community was positively associated with both the average number of HP donor species and the number of CP grains found on stigmas, suggesting a common underlying cause. Indeed, multispecies assemblages of co-flowering plants can increase pollinator attraction by increasing (1) the number and variety of flowers present in a patch^16,32,33,35,36,69,70^, (2) resource diversity^71^, (3) chromatic or color diversity^34,72^, and (4) the likelihood of including a “magnet” species^37^; hence promoting inter and intraspecific pollen transfer. Even though the receipt of CP increased monotonically with plant species richness, this rise saturates with the increase in the number of plant species in the community (Fig. 4). This latter decelerated increase in CP receipt could indicate functional redundancy in the positive effect of plant diversity when it is measured in taxonomic terms^73^.

## Conclusions

Plant–plant facilitation has shown to be key in promoting plant diversity worldwide^5,47,48^, the present study further provides evidence that this process can be bidirectional since plant diversity can also promote facilitation. Despite its correlative nature, this study represents a step forward towards a deeper understanding of the positive effects of plant diversity on pollination in environments subjected to abiotic stress. Overall, our findings show that the deposition of CP is related positively to HP donor diversity on stigmas, the latter reflecting the species diversity of the surrounding community. Therefore, our results strongly suggest that plant diversity boosts pollination success in high-Andean plant communities, which is crucial for the sexual reproduction of alpine plants. This diversity effect could be especially critical in plant communities experiencing chronic pollination limitation, as the ones dominated by outcrossing species that characterize these harsh environments^59,74^. In a previous work, we revealed how diversity begets diversity in these communities via direct facilitative interactions, whose effects scale up from the local to the regional scale^49^. Here, we show that this positive diversity feedback can also involve indirect, pollinator-mediated, plant–plant interactions.

## Methods

### Study sites and sampling

Field work was conducted in the alpine zone (> 1500 m), above the *Nothofagus pumilio* timberline, of the Andean mountains of northwestern Patagonia, Nahuel Huapi National Park, Argentina. Maximum mountain elevation in this region is about 2200 m with just a few peaks above 3000 m. Mean annual temperature in the alpine region is 3.7 °C, with short, mild, dry summers and cold, wet winters. Mean annual precipitation is approx. 860 mm, mostly in the form of snow between May and October^49^. In these high-Andean communities, plant cover is low (< 20% at 1600 m vs. < 5% at 2000 m) and vegetation is sparse, resulting in a patchy-landscape represented by isolated cushion-plant patches and a surrounding bare ground matrix^49,75^. Despite this low plant cover, the Andean flora of the region includes more than 250 species of vascular plants, with Asteraceae, Poaceae and Apiaceae being the best represented angiosperm families^76^. Overall, predominant life-forms comprise perennial herbs, shrubs and cushion plants. High-Andean Patagonian plant communities are characterized by highly pollinator-dependent breeding systems, including relatively high incidences of self-incompatibility and dioecism^59,77^. Pollen-supplementation assays carried out in four species suggests that pollination frequently limits reproduction in these Andean plant communities (S. Gavini, unpublished data). Bees, most notably the introduced alien bumble bee *Bombus terrestris* (Apidae), which replaced the native bumble bee *B. dahlbomii*, flies (mainly Syrphidae and Nemestrinidae), and butterflies (Nymphalidae and Pieridae) are the most common pollinators (authors personal observation; see also^57,58,78^].

Animal-pollinated plant species from the high-Andean alpine environment, identified based on previous field observations of legitimate flower visits (i.e., those in which insects contacted the flower’s sexual parts) and patterns of interspecific pollen transfer^7^ were sampled at three altitudes (about 1600, 1800 and 2000 m) on each of three mountains: (a) the north face of Cerro Challhuaco (41° 26′ 6″ S, 71° 31′ 9″ W), (b) the east face of Cerro Catedral (41° 17′ 1″ S, 71° 48′ 6″ W), and (c) the east face of Cerro López (41° 10′ 1″ S, 71° 57′ 3″ W) during the 2010–2011 austral flowering season (from December to March). The plant community at each altitudinal level-mountain combination was sampled once every two weeks during the study period (from four to six times in total per community). The three study communities occurring in each mountain were sampled on the same date or over two consecutive dates. In each community and on each sampling day, all animal-pollinated flowering plant species in bloom were identified in a 100 × 25 m permanent transect (Table S1). For each species within each transect, five plant individuals were randomly selected, whenever possible, and five senescent flowers (i.e., post-anthesis) were collected per individual, and stored separately in clean eppendorf tubes in 70% ethanol. All experimental research and field studies on plants (either cultivated or wild), including the collection of plant material, comply with relevant institutional, national, and international guidelines and legislation, as established by the National Park Administration of Argentina. Later in the laboratory, each stigma was mounted on a slide and stained with Alexander’s solution^79^. Each sample was analyzed under microscope at 400× and all conspecific and heterospecific pollen grains on the stigma were counted. The identity of the grains was verified using photographs of pollen grains of all plant species at each study site^7^. Further methodological details can be found in the Supplementary Material of Tur et al.^7^.

### Statistical analyses

Data from pollen deposition on single stigmas were used to fit generalized linear mixed-effects models (GLMMs) for estimating (1) the overall relation of the number of conspecific pollen (CP) grains to the number of heterospecific pollen (HP) donor species (i.e., HP richness), and (2) the sign of this relation (positive, neutral or negative) for each receptor plant species. All analyses were performed in the R software version 3.4.4^80^, using the *glmmTMB* function in R package glmmTMB version 0.2.3^81^. Number of CP grains per stigma was the response variable in all models, and a negative binomial distribution of errors (NBD) was applied to account for observed overdispersion in data counts. Specifically, we considered a NBD of type “1” where the variance increases linearly with the mean, i.e. variance = mu*(1 + phi).

#### Overall relation of conspecific pollen receipt to multispecies heterospecific loads

The model for the estimation of the overall effect included HP richness (as a numeric variable, ranging from 0 to 6), altitude (as a categorical variable with three levels, i.e. 1600, 1800 and 2000 m), and the interaction between HP richness and altitude as fixed predictors. Moreover, we included two other important predictors as covariates: (1) sampling fortnight (as a factor with seven levels), which was considered as a surrogate variable to account for changes in plant and pollinator densities over the flowering season; and (2) total abundance of HP (i.e., total number of HP grains pooled irrespective of donor species), which allowed us to estimate the relation of CP grains per stigma to HP pollen richness independent of any effect related to the abundance of HP (for more details see “Supporting Information”). We performed a Variance Inflation Factor (VIF) analysis because richness and abundance of HP were positively correlated overall (*r* = 0.62, *P* < 0.001), which might cause collinearity that could lead to misleading results when both predictors are included in the model^82^. Although much divergence exists in the literature regarding the VIF value-threshold that determines problematic collinearity^82,83^, VIF value for our model was 1.64, far below the lowest critical threshold limit of 3.3^82^. Because collinearity issues were not of concern in this study, we were able to compare the magnitude of the effect of the HP richness independent of its absolute abundance. To deal with potential pseudoreplication derived from clustering hierarchically-structured data^84^, we included “plant individual” nested within “receptor species” and “community” nested within “mountain” as crossed random intercept effects. To compare the relative strength of the effect of HP richness with that of the effect of HP abundance, we re-ran this model with these two variables standardized as $({x}_{i}-\overline{x })/\mathrm{SD}$, where ${x}_{i}$ is each individual observation (either for HP richness or abundance), and $\overline{x }$ and SD are the mean and standard deviation of each of the two variables.

#### Species-specific responses to multispecies heterospecific loads

For the second objective, i.e. determining for each species the sign of the relation between HP richness and CP deposition, a separate model was fitted for each of the nine alpine communities (i.e., 3 mountains × 3 altitudes) with HP richness as a fixed predictor, and sampling date and HP abundance as covariates. As before, a VIF analysis was performed using the data subset for each community and, again, collinearity was low with VIF values ranging from 1.42 to 2.51. Models included “plant individual” nested within “receptor species” as a random effect, but in this case considering a varying-intercept and varying-slope models^85^, because the response of each receptor species to HP richness might be different (see “Supporting Information”). Following the conceptual framework of Tur et al*.*^7^, the slope estimated for each species (which, unlike Tur et al*.,* here it shows the relationship between CP quantity and HP richness, independent of HP abundance) was considered an indicator of the effect of pollinator sharing from the perspective of the receptor species as related to the extent of multispecies visitation. Because of the interval defined by a statistical estimate ±  ~ 2× standard error (SE) approximates to a 95% confidence interval^85^, a slope ± 2SE > 0 was considered evidence of an overall facilitative effect, a slope ± 2SE overlapping with 0 of a neutral effect, and slope ± 2SE < 0 of a competitive effect of multispecies visitation^7^. The percentage of receptor species experiencing each type of effect was determined for each community and compared across altitudes with a chi-square test.

#### Relation of CP and HP on stigmas to community species richness

Lastly, we studied the relation between pollen deposition on stigmas (both HP richness and CP quantity) and plant species richness in the community. Total number of plant species in bloom and total number of different HP donors were calculated for each mountain, altitude and sampling date combination (n = 44). We first explored whether the cumulative observed number of heterospecific donors across all stigmas sampled in a given community at a given time reflected the potential number of pollen donors (i.e., sampling completeness)^86^. This was done by comparing the observed number of distinctive HP donors with the non-parametric asymptotic estimate of the rarefaction curve of the cumulative number of HP donor species with the number of sampled stigmas for each of the 44 community × time combinations. We used the Chao-2 asymptotic value as the expected number of HP donor species because it is the most proper estimator of species richness for incidence data^87^. After proving that the observed number of total pollen donors correlated closely with the number of potential donor species (see “Results”), we assessed whether the former variable was related to the blooming plant species richness in the community by fitting a GLMM with a Poisson error distribution (no overdispersion was detected). In this analysis, we considered the total number of heterospecific donors as the response variable and the (log-transformed) number of species in bloom as predictor, including “community” nested within “mountain” as a random effect (random intercepts).

We also compared the similarity in the composition of the assemblage of heterospecific donor species found on stigmas with the co-flowering species pool present in the community at a given time. Accordingly, we calculated beta diversity by means of the Sørensen index (βsor), which actually measures dissimilarity between species assemblages, varying between 0, when the two assemblages share the same species pool, to 1, when the two assemblages do not have any species in common^88^. Following the method proposed by Baselga and Orme^89^, we partitioned total pairwise dissimilarity into species turnover (βsim, Simpson Index) and species loss (βnes, nestedness). We calculated beta diversity and its components using the function beta.pair in the package betapart^88,89^ for each mountain, altitude and sampling date combination. Overall dissimilarity (donor species on stigmas vs. co-flowering species in the community) was analyzed with a linear mixed-effects model (LMM) (i.e., Gaussian error distribution) using altitude as a fixed factor, and mountain and sampling date as random effects.

Finally, we fitted LMMs with Gaussian error distributions to analyze whether mean richness of HP and mean number of CP deposited per stigma (response variables) were related to the number of species in bloom in the community (predictor). For these two analyses, mean values of HP and CP were calculated for each plant species within each mountain, altitude and sampling date combination (n = 398). Both response variables and predictor were log-transformed in order to meet model assumptions and increase model fit, respectively. Models included “community” nested within “mountain” as a random effect (random intercepts only). Predicted values with 95% confidence intervals of these models were computed and plotted with the function *ggpredict* of the ggeffects and ggplot2 package in the R software version 3.4.4^80^.

### Ethical approval

All corresponding permits were obtained from National Park Administration of Argentina for the realization of this study and the collection of plant material.

## Supplementary Information

Supplementary Information.

## Data Availability

Data deposited at Zenodo: https://doi.org/10.5281/zenodo.5553309.
