# Supplementary material for: Pollination success increases with plant diversity in high-Andean communities
Source: Sci Rep. 2021 Nov 11;11:22107. doi: 10.1038/s41598-021-01611-w (PMC8586006; doi:10.1038/s41598-021-01611-w)
Supplement: Supplementary file 1 — Supplementary Information. [file 41598_2021_1611_MOESM1_ESM.pdf]

Supporting Information

Pollination success increases with plant diversity in high-Andean communities

Sabrina S. Gavini, Agustín Saez, Cristina Tur and Marcelo A. Aizen

Table S1: List of plant species and their occurrence (marked with an X) in the nine study communities.

			CATEDRAL			CHALLHUACO			LÓPEZ		
**Species**	**Family**	**Abbreviation**	**1600**	**1800**	**2000**	**1600**	**1800**	**2000**	**1600**	**1800**	**2000**
Adesmia corymbosa	Fabaceae	Ade.cor				X	X			X	X
Adesmia glomerula	Fabaceae	Ade.glo	X								
Adesmia longipes	Fabaceae	Ade.lon	X						X	X	
Adesmia parviflora	Fabaceae	Ade.par		X			X				
Anagallis alternifolia	Primulaceae	Ana.alt							X		
Anemone multifida	Ranunculaceae	Ane.mul				X	X				
Arjona patagonica	Schoepfiaceae	Arj.pat					X				
Armeria maritima	Plumbaginaceae	Arm.mar			X	X	X				X
Azorella andina	Apiaceae	Azo.and						X			
Azorella monantha	Apiaceae	Azo.mon				X					
Azorella nivalis	Apiaceae	Azo.niv	X	X	X	X	X				
Azorella prolifera	Apiaceae	Azo.pro				X		X			
Berberis empetrifolia	Berberidaceae	Ber.emp	X								
Calceolaria filicaulis	Calceolariaceae	Cal.fil	X		X				X	X	
Cardamine cordata	Brassicaceae	Car.cor		X						X	
Cerastium arvense	Caryophyllaceae	Cer.arv	X	X	X		X	X	X		X
Chiliotrichum diffusum	Asteraceae	Chi.dif	X	X		X	X				
Discaria chacaye	Rhamnaceae	Dis.cha	X	X		X					
Draba gilliesii	Brassicaceae	Dra.gil		X				X			
Epilobium australe	Onagraceae	Epi.aus	X		X						
Erigeron leptopetalus	Asteraceae	Eri.lep	X	X	X		X	X	X		X
Escallonia alpina	Escalloniaceae	Esc.alp		X					X		
Euphrasia meiantha	Orobanchaceae	Eup.mei	X	X					X	X	

Gamocarpha scapigera	Calyceraceae	Gam.sca				X					
Gamocarpha selliana	Calyceraceae	Gam.sel				X	X				
Gaultheria caespitosa	Ericaceae	Gau.cae								X	X
Gaultheria pumila	Ericaceae	Gau.pum	X	X	X	X	X	X	X	X	X
Geranium sessiliflorum	Geraniaceae	Ger.ses					X		X		
Hypochaeris tenuifolia	Asteraceae	Hyp.ten	X	X		X	X				
Leucheria millefolium	Asteraceae	Leu.mil		X		X	X				
Moschopis caleofuensis	Calyceraceae	Mos.cal				X	X				
Nassauvia aculeata	Asteraceae	Nas.acu				X	X				
Nassauvia darwinii	Asteraceae	Nas.dar					X				
Nassauvia dentata	Asteraceae	Nas.den								X	
Nassauvia pulcherrima	Asteraceae	Nas.pul			X						X
Nassauvia pygmaea	Asteraceae	Nas.pyg			X			X			X
Nassauvia revoluta	Asteraceae	Nas.rev			X			X			X
Ochetophila nana	Rhamnaceae	Och.nan	X	X			X				
Oreopolus glacialis	Rubiaceae	Ore.gla		X	X	X	X				
Ourisia alpina	Plantaginaceae	Our.alp		X					X	X	
Ourisia breviflora	Plantaginaceae	Our.bre								X	
Ourisia ruellioides	Plantaginaceae	Our.rue							X		
Oxalis adenophylla	Oxalidaceae	Oxa.ade		X	X						
Oxalis erythrorhiza	Oxalidaceae	Oxa.ery			X			X			
Perezia bellidifolia	Asteraceae	Per.bel	X			X	X		X		
Perezia delicata	Asteraceae	Per.del							X		
Perezia fonkii	Asteraceae	Per.fon		X					X		
Perezia pilifera	Asteraceae	Per.pil		X							
Perezia recurvata	Asteraceae	Per.rec					X				
Perezia sp.	Asteraceae	Per.sp							X	X	
Phacelia secunda	Boraginaceae	Pha.sec	X			X					

Pinnasa nana	Loasaceae	Pin.nan		X	X	X	X	X			
Polygala salasiana	Polygalaceae	Pol.sal		X		X	X				
Quinchamalium chilense	Schoepfiaceae	Qui.chi	X	X		X	X		X	X	
Ranunculus peduncularis	Ranunculaceae	Ran.ped								X	X
Senecio argyreus	Asteraceae	Sen.arg	X	X		X	X		X		
Senecio baccharidifolius	Asteraceae	Sen.bac		X			X				
Senecio boelckei	Asteraceae	Sen.boe						X			
Senecio crithmoides	Asteraceae	Sen.cri			X	X	X	X	X	X	X
Senecio diemii	Asteraceae	Sen.die		X							
Senecio fistulosus	Asteraceae	Sen.fis	X								
Senecio gnidioides	Asteraceae	Sen.gni					X				
Senecio hieracium	Asteraceae	Sen.hie	X	X					X	X	X
Senecio pachyphyllos	Asteraceae	Sen.pac			X			X			X
Senecio peteroanus	Asteraceae	Sen.pet					X				
Senecio poeppigii	Asteraceae	Sen.poe	X	X			X		X	X	
Senecio portalesianus	Asteraceae	Sen.por			X			X		X	X
Senecio sp. *1*	Asteraceae	Sen.sp1	X	X							
Senecio sp. *2*	Asteraceae	Sen.sp2		X							
Senecio sp. *3*	Asteraceae	Sen.sp3				X	X				
Senecio sp. *4*	Asteraceae	Sen.sp4								X	
Senecio sp. *5*	Asteraceae	Sen.sp5								X	
Senecio sp. *6*	Asteraceae	Sen.sp6								X	
Senecio subdiscoideus	Asteraceae	Sen.sub		X							X
Senecio trifurcatus	Asteraceae	Sen.trif							X	X	
Senecio triodon	Asteraceae	Sen.trio		X							
Silene andicola	Caryophyllaceae	Sil.and							X	X	
Sisyrinchium arenarium	Iridaceae	Sis.are	X				X				
Symphyotrichum glabrifolium	Asteraceae	Sym.gla							X	X	

Trifolium repens	Fabaceae	Tri.rep							X		
Unidentified species	Asteraceae	Unk.sp				X					
Valeriana carnosa	Caprifoliaceae	Val.car				X	X				
Valeriana fonckii	Caprifoliaceae	Val.fon								X	
Valeriana macrorhiza	Caprifoliaceae	Val.mac		X							
Valeriana philippiana	Caprifoliaceae	Val.phi								X	
Valeriana sp.	Caprifoliaceae	Val.sp		X							
Vicia bijuga	Fabaceae	Vic.bij					X				
Viola columnaris	Violaceae	Vio.col	X								
**TOTAL**	**27**	**88**	**24**	**33**	**17**	**26**	**34**	**14**	**24**	**25**	**15**

Figure S1: Percentage of stigmas in each category of HP richness (i.e., number of HP donor species per stigma) for each of the nine study plant communities (three mountains x three altitudes). The value in the top right corner of each graph represents the total number of stigmas sampled in each community.

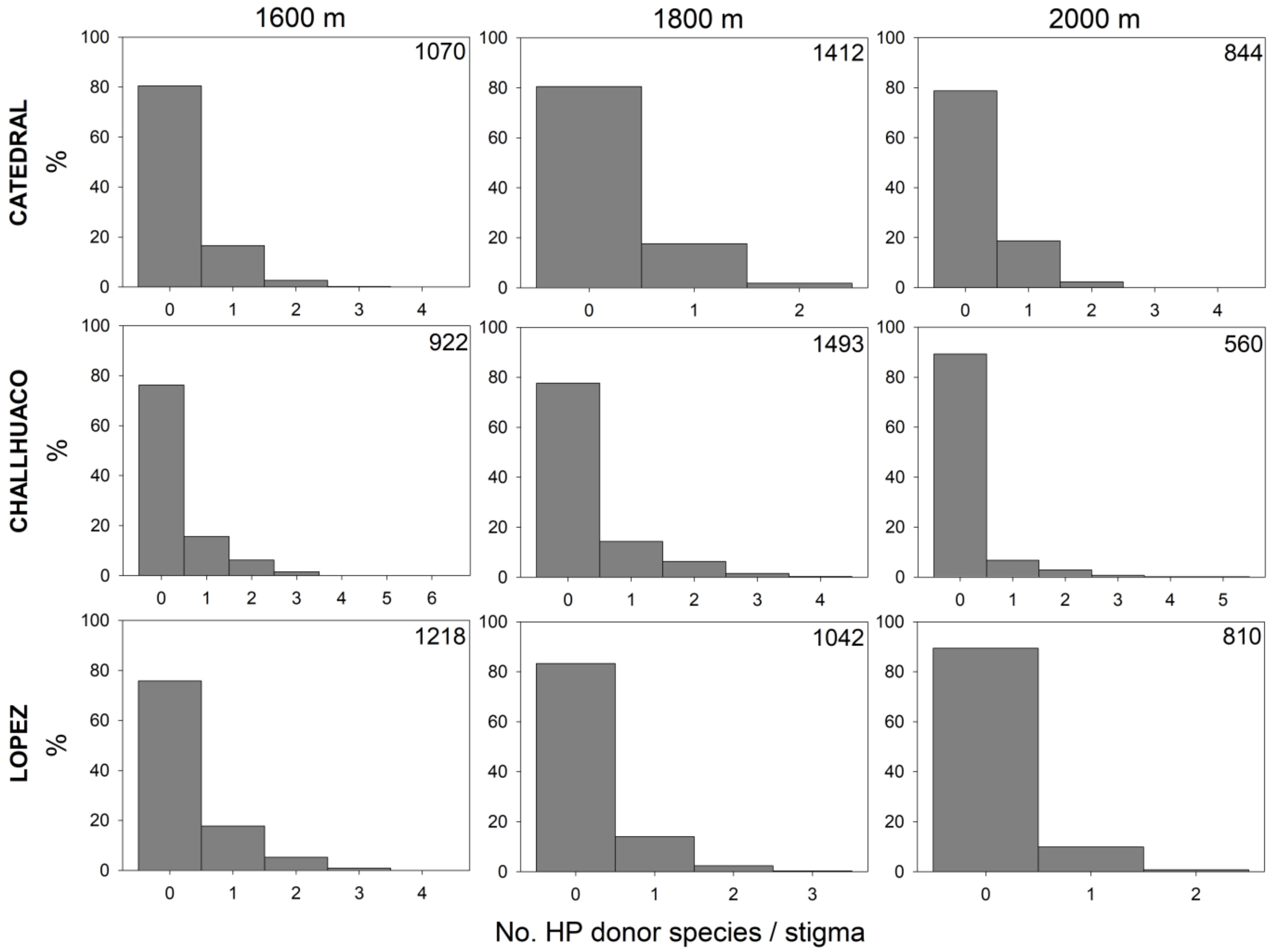

Table S2: Results of the generalized linear mixed effect model assessing the overall effect of HP richness, altitude and their interaction on the receipt of CP on stigmas, considering sampling fortnight (time) and HP abundance as covariates.

	χ^2	df	P
**Intercept**	144.13	1	2.2×10^{-16} ***
**HP richness**	64.10	1	1.18×10^{-15} ***
**Altitude**	2.58	2	0.275
**HP richness x Altitude**	4.28	2	0.117
**Time**	31.86	6	1.74×10^{-5} ***
**HP abundance**	8.85	1	0.0029 **

Figure S2: (A) Accumulation curves of the heterospecific pollen donor species as a function of the sampling effort (i.e., number of stigmas) for the 44 combinations of community x sampling time. (B) Correlation between the expected number of heterospecific pollen donor species (Chao-2 asymptotic estimate of each curve) and the observed number of heterospecific pollen donor species.

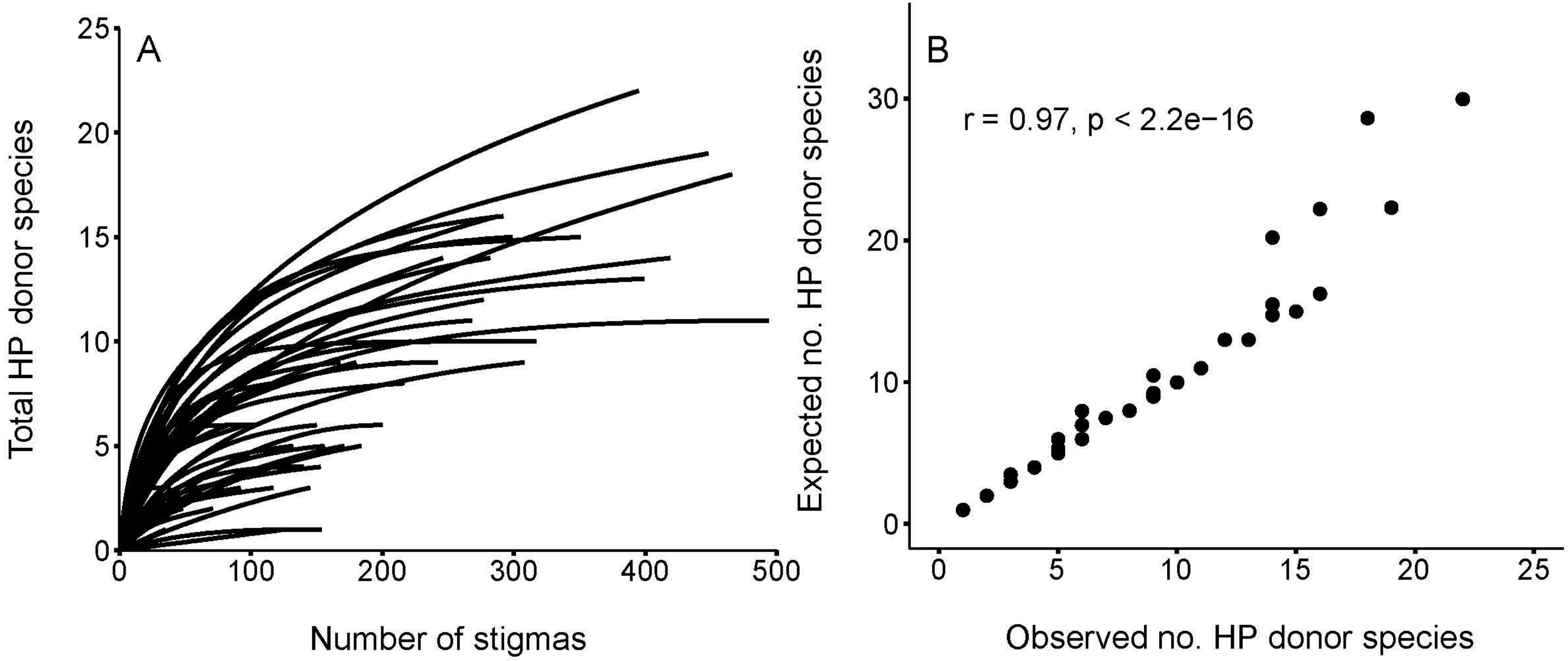

Table S3: Pairwise comparisons of species composition between the cumulative species pool whose pollen was found on stigmas of other species (i.e., HP donor species) and the flowering community at a given time expressed as beta diversity or dissimilarity. Dissimilarity was partitioned following the framework of Baselga (2010) as βsor = βsim + βnes, where βsor (Sorensen index) represents the total difference in species composition between assemblages owed to βsim or species turnover (Simpson index) and βnes or species loss (nestedness-driven dissimilarity). Mean values (±1SE) were obtained averaging dissimilarities across sampling fortnights within each community.

**Mountain**	**Altitude**	**Beta diversity βsor**		**Turnover βsim**		**Nestedness βnes**	
		mean	±1 SE	mean	±1 SE	mean	±1 SE
**Catedral**	1600	0.261	0.055	0.105	0.050	0.156	0.060
	1800	0.292	0.070	0.129	0.085	0.163	0.055
	2000	0.322	0.065	0.282	0.090	0.040	0.030
**Challhuaco**	1600	0.147	0.060	0.096	0.040	0.052	0.050
	1800	0.420	0.120	0.331	0.150	0.090	0.040
	2000	0.564	0.090	0.243	0.150	0.321	0.180
**López**	1600	0.182	0.054	0.085	0.040	0.097	0.035
	1800	0.212	0.031	0.097	0.065	0.115	0.042
	2000	0.430	0.150	0.356	0.160	0.074	0.020

Figure S3: Contribution of turnover (βsim, Simpson index) and species loss or nestedness (βnes) to the total dissimilarity or beta diversity (βsor) between the assemblage of plant species whose pollen was found on heterospecific stigmas (i.e., HP donor species) and the species present in the community.

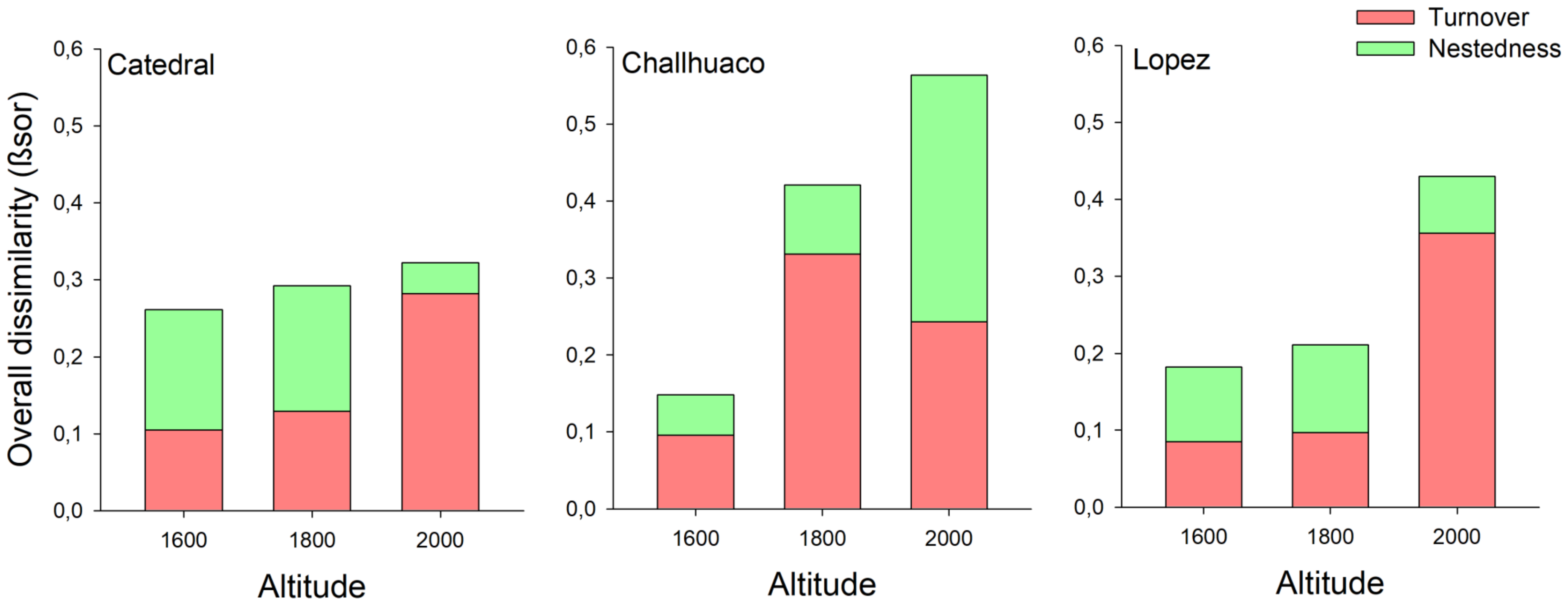

Details on models for the estimation of diversity-mediated effects

We used varying-intercept and/or varying-slope multilevel generalized linear models. Such multilevel modelling approach allows for reliable estimates, while accounting for the inherent clustered structure of the data (Gelman & Hill, 2007). Models were performed with the package glmmTMB v0.2.3 (Magnusson *et al.,* 2017).

Model 1, for the estimation of the overall quantitative effect of heterospecific pollen richness on conspecific pollen receipt, was a Negative Binomial GLMM as follows:

CP ~ HP Richness *Altitude + HP Abundance + Time +

(1 **|** Species / Plant) + (1 **|** Mountain / Community)

CP is the number of conspecific pollen grains on stigma, **HP Richness** the number of heterospecific pollen donor species on stigma, **Altitude** is a factor that encompasses three elevations (1600 m, 1800 m, and 2000 m), the covariate **HP Abundance** is the total number of heterospecific pollen grains irrespective of donor species, and the covariate **Time** (sampling fortnights) accounted for changes in plant and pollinator densities over the flowering season. **Plant** nested within **Species** and **Community** nested within **Mountain** are included as crossed random intercept effects.

Model 2, for the estimation of the species-specific responses of heterospecific pollen richness on conspecific pollen receipt, was a Negative Binomial GLMM as follows:

CP ~ HP Richness + HP Abundance + Time +

(1 + HP Richness | Species / Plant)

The fitted model is a varying-intercept and varying-slope model, for each species the model estimate a different intercept and slope between CP receipt and HP richness.
